# An Uncommon Case of Myocarditis Secondary to Durvalumab Plus Tremelimumab

**DOI:** 10.7759/cureus.43628

**Published:** 2023-08-17

**Authors:** Porfirio E Diaz-Rodriguez, Claudia M Muns-Aponte, Sharolyn I Velazquez-Acevedo, Cristina M Ortiz-Malave, Jose Acevedo, Francisco G Merced-Ortiz

**Affiliations:** 1 Cardiology, Veterans Affairs Medical Center, San Juan, PRI; 2 Internal Medicine, Veterans Affairs Medical Center, San Juan, PRI; 3 Internal Medicine, Veterans Affairs Caribbean Healthcare System, San Juan, PRI; 4 Cardiology, Veterans Affairs Caribbean Healthcare System, San Juan, PRI

**Keywords:** fulminant myocarditis, durvalumab, tremelimumab, ekg, ventricular tachycardia, complete av block, immune check-point inhibitor, myocarditis

## Abstract

Tumor immunotherapy is an important clinical strategy for the treatment of various solid and hematological malignancies, and its use is on the rise. Immune checkpoint inhibitors (ICIs) are immunotherapies that boost anticancer immune responses by targeting receptors on the surface of T-lymphocytes. Two important ICIs are anti-programmed death ligand-1 (anti-PD-L1) monoclonal antibodies and anti-cytotoxic T-lymphocyte-associated antigen-4 (anti-CTLA-4) monoclonal antibodies. Tremelimumab (anti-CTLA-4) and durvalumab (anti-PD-L1) have been shown to be effective monotherapies. However, their combination has demonstrated effective and encouraging antitumor activity with manageable safety in patients with unresectable hepatocellular carcinoma. We present the case of an 80-year-old male with hepatocellular carcinoma who had undergone drug-eluting bead transarterial chemoembolization (DEB-TACE) on three occasions and had been started on a combination of ICIs, durvalumab, and tremelimumab. He subsequently developed various immune-related adverse effects in different organ systems, including hepatic and cardiovascular complications. Appropriate treatment was administered, but ultimately, he passed away. We aim to discuss the initial evaluation for suspected immune-related adverse events, specifically those related to myocarditis and its various manifestations, prognosis, and treatment.

## Introduction

Tumor immunotherapy is an important clinical strategy for the treatment of various solid and hematological malignancies [1]. Immune checkpoint inhibitors (ICIs) are immunotherapies that boost anticancer immune responses by targeting receptors on the surface of T-lymphocytes [2]. In other words, they redirect the host immune system toward fighting off tumor cells. Two important ICIs are anti-programmed death ligand-1 (anti-PD-L1) monoclonal antibodies and anti-cytotoxic T-lymphocyte-associated antigen-4 (anti-CTLA-4) monoclonal antibodies. Tremelimumab (anti-CTLA-4) and durvalumab (anti-PD-L1) have been shown to be effective monotherapies. However, their combination has demonstrated effective and encouraging antitumor activity with manageable safety in patients with unresectable hepatocellular carcinoma [3]. Nevertheless, due to their mechanism of action, ICIs can lead to adverse events in almost any organ system. Studies are still being developed to identify new strategies for the prevention and appropriate management of these known complications.

Autoimmune toxicities due to the activation of more responsive T cells result in the occurrence of immune-related adverse events (IRAEs). Overall, about 60-80% of patients who receive ICI therapy experience IRAEs [4]. Although rare, fulminant and fatal toxic effects, such as hepatitis and cardiotoxicity, have been described [4]. Cardiovascular-related events may present as arrhythmias, pericardial disease, vasculitis, myocardial infarction, and Takotsubo cardiomyopathy, with the most common being myocarditis [2]. A retrospective study of data from eight clinical centers found that the prevalence of myocarditis was 1.14% [5]. The pathophysiological mechanism of how ICI myocarditis is caused is not fully understood, but infiltration of T cells into the injured myocardial tissue was described in two cases of myocarditis in patients treated with a combination of ipilimumab (anti-CTLA-4) and nivolumab (anti-PD-1) [6]. Most cases occur early, with a median time to the onset of toxicity of 30 days, usually after the first or second ICI infusion [7]. Additionally, ICI-related myocarditis can lead to cardiogenic shock, multiorgan failure, ventricular arrhythmias, and even death in severe cases [2].

## Case presentation

**Figure 1:**
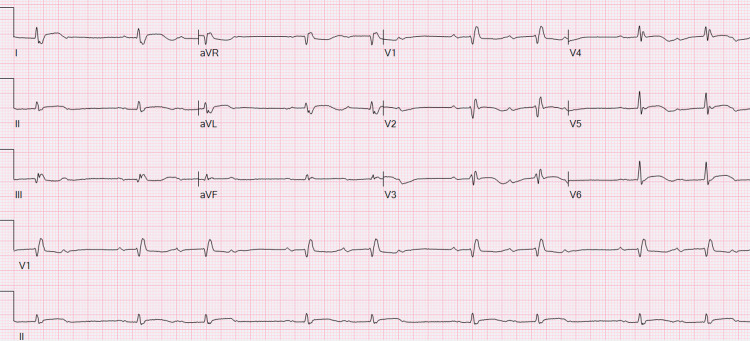
Admission ECG showing sinus bradycardia with second-degree AV block type 1 (Wenckebach), right bundle branch block, and low voltage. ECG: electrocardiogram; AV: atrioventricular.

**Figure 2:**
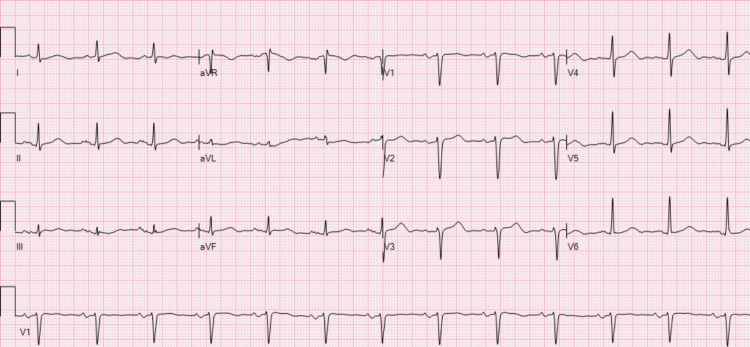
Baseline ECG showing normal sinus rhythm with no conduction abnormalities. ECG: electrocardiogram.

An 80-year-old male with a history of paroxysmal atrial fibrillation on rivaroxaban and hepatocellular carcinoma had undergone drug-eluting bead transarterial chemoembolization (DEB-TACE) on three occasions with follow-up imaging that showed an appropriate response to therapy. He had been started on a combination of ICIs, namely durvalumab and tremelimumab, just 22 days before the presentation. He arrived at the ER after experiencing worsening shortness of breath, myalgias, and general malaise. Dyspnea improved after the administration of a respiratory therapy combination of albuterol, ipratropium, and dexamethasone. However, elevated liver enzymes with aspartate transaminase (AST) of 646 U/L and alanine transaminase (ALT) of 201 U/L (baseline was normal at 27 and 18 U/L) were detected on initial labs, leading to his admission for observation and management of suspected ICI-induced liver injury. He was started on 60 mg of prednisone per os daily. One day later, the patient developed respiratory distress, which required non-invasive mechanical ventilation. An electrocardiogram (ECG) showed a Mobitz type 1 atrioventricular (AV) block with a right bundle branch block (Figure 1), which was new when compared to the baseline (Figure 2). 

**Figure 3:**
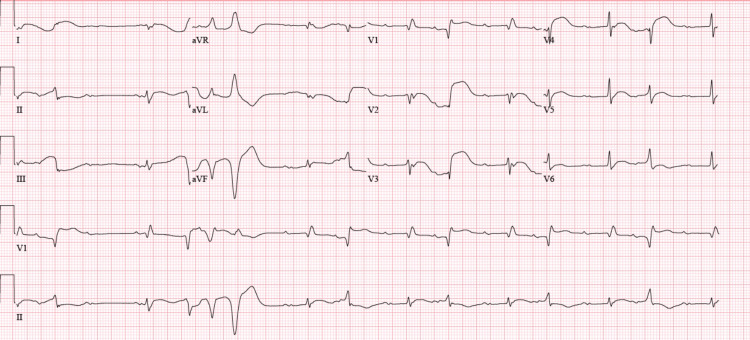
ECG performed two days after admission to the ICU. This ECG shows a sinus rhythm with complete AV block, junctional escape rhythm with right bundle branch block, one episode of non-sustained VT, and increased PVC burden in the bigeminal pattern. Manually corrected QT was increased at 522 ms. ECG: electrocardiogram; ICU: intensive care unit; AV: atrioventricular; PVC: premature ventricular contraction; VT: ventricular tachycardia.

**Figure 4:**
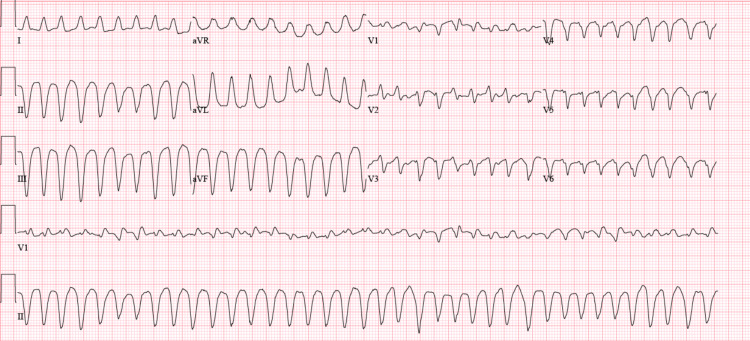
ECG taken shortly after a marked PVC burden. After noticing the complete atrioventricular block with increased PVC burden, the patient developed one episode of Torsade de Pointes. ECG: electrocardiogram; PVC: premature ventricular contraction.

High-sensitive troponins were markedly elevated at 1,640 ng/L (abnormal cut-off >22 ng/L) in the absence of chest pain and a lack of ST segment changes suggestive of acute coronary syndrome. Pro-B-type natriuretic peptide (BNP) level was 824 pg/mL (range 0-450 pg/mL). He was transferred to the intensive care unit for a higher level of care, where a bedside echocardiogram demonstrated a preserved ejection fraction (EF) of 50-55% with no valvulopathies or wall motion abnormalities. During his second day in the ICU, the patient became hemodynamically unstable, requiring vasopressor therapy. Right-sided ptosis and rightward disconjugate gaze were observed on physical examination, for which myasthenia-like syndrome was also suspected. The patient had a negative inspiratory force of 14, for which invasive mechanical ventilation was started. He began to develop electrical disturbances that led to an increased premature ventricular contraction (PVC) burden and complete atrioventricular block (CAVB) (Figure 3). Soon after, he developed runs of non-sustained ventricular tachycardia (VT) with subsequent Torsade de Pointes (Figure 4). Potassium was at 4.7 mEq/L and magnesium was at 1.8 mg/dL. There was no evidence of ST segment elevations on the ECG, but manually corrected QT was increased at 522 ms. Intravenous amiodarone was initiated along with high-dose steroid pulsation therapy for three days (1 g/kg) and mycophenolate mofetil for additional immunosuppression. A transvenous pacemaker was immediately placed for overdrive pacing and to avoid further QT prolongation and suppress PVCs. A new bedside echocardiogram was performed, which showed severe left ventricle dysfunction with an EF of 25-30%. An endomyocardial biopsy was considered but not performed due to hemodynamic instability and ongoing anticoagulation with rivaroxaban.

**Figure 5:**
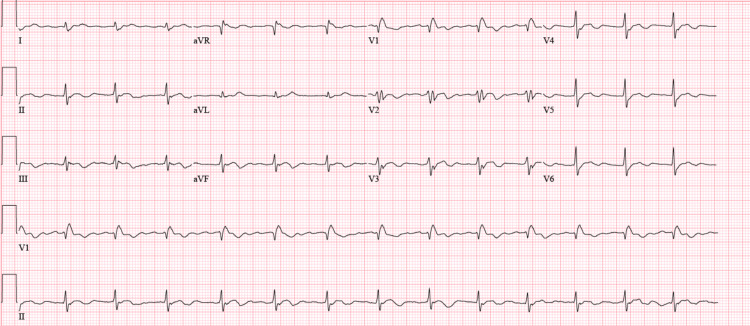
ECG with the return of 1:1 sinus conduction. A few days after starting steroid and immunosuppressive therapy, the patient regained 1:1 sinus conduction. ECG shows normal sinus rhythm with first-degree AV block, right bundle branch block, diffuse T-wave inversions, and prolonged QT at 507 ms. ECG: electrocardiogram; AV: atrioventricular.

Slow, yet notable, clinical improvement was observed in the following days after starting immunosuppressive therapy. EF increased close to preserved function, troponin levels were in a decreasing trend, there was also electrocardiographic improvement with the return of AV conduction (Figure 5), and his myasthenia-like symptoms mildly improved.

However, the patient developed multiple fever spikes and was started on empiric broad-spectrum antibiotic therapy with vancomycin and cefepime. Sputum culture was eventually reported positive for methicillin-resistant *Staphylococcus aureus*. Despite aggressive therapy, the patient continued to deteriorate due to septic shock and ultimately died after 14 days of hospitalization after the withdrawal of life support as per family wishes.

## Discussion

ICIs are monoclonal antibodies that target the host immune negative regulation receptors. They specifically target receptors on T cells, which are the key to inhibitory pathways that block antitumor cell activation [4]. Indications for their use in cancer treatment continue to expand, but it is important to note that uncontrolled activation of cytotoxic T cells has brought about many side effects in clinical immunotherapy. Since ICIs activate immunity throughout the body, not just tumor cells, IRAEs can involve almost all organ systems. In parallel with their increased use, recognition of IRAEs has also improved [4]. While some adverse effects are treatable with low-dose steroids, others can quickly lead to life-threatening complications if not recognized early in their development [8]. ICI-related myocarditis has a reported incidence of 0.04-1.14%, but when compared with other IRAEs, it has a significantly higher associated mortality of 25-50% [9]. Additionally, the incidence and mortality of immune-related myocarditis tend to be higher with combination therapy than with ICI monotherapy [9].

The mechanism behind ICI-induced myocarditis is unknown. However, studies involving histopathological analysis of affected tissue suggest that T cells could be targeting an antigen shared by the tumor, skeletal muscle, and the heart, or the same T-cell receptor may be targeting a tumor antigen and a different but homologous muscle antigen [6]. The American Society of Clinical Oncology has proposed a grading system for the different cardiovascular manifestations that can vary from asymptomatic elevation of cardiac biomarkers to life-threatening disease with cardiac study abnormalities (grades 1-4). All grades warrant workup and intervention given the potential for cardiac compromise. Physicians should hold and permanently discontinue ICI therapies in patients with any grade of complications, and they should promptly initiate high-dose corticosteroids (1-2 mg/kg of prednisone). If there is no response to initial steroids, one should consider increasing steroid doses to those used in cardiac transplant (1 g methylprednisolone daily) with the addition of mycophenolate, infliximab, or antithymocyte globulin (ATG) [9]. The presentation of cardiovascular complications of checkpoint inhibitors could include arrhythmia, palpitations, chest pain, or signs and symptoms of heart failure (shortness of breath, peripheral edema, pleural effusion, fatigue). Also, many patients present with other IRAEs, such as myositis and myasthenia‐like syndrome, which can cause overlapping symptoms, making prompt diagnosis increasingly challenging [9].

The first step in diagnosing ICI-induced myocarditis is to rule out more common cardiac problems with similar presentations, such as acute coronary syndrome, chronic ischemic heart disease with or without heart failure, or other causes of non-ischemic heart failure. In a clinical case series of 35 patients with ICI‐related myocarditis from multiple institutions, 94% presented with elevated troponin levels. BNP, reported in 66% of cases, is also one of the most common initial lab factors that suggest possible myocarditis. This study also revealed that patients with significantly elevated cardiac enzymes were more likely to develop MACE, serving as guidance in prognosis [6]. In the appropriate clinical setting, ECG findings, such as new prolongation of the PR interval, AV block, ventricular arrhythmias, frequent premature ventricular complexes, ST depression, or diffuse T-wave inversions, support the diagnosis. In the case of our patient, the ECG initially showed a new-onset Mobitz type 1 AV block with a right bundle branch block, and high-sensitive troponins were markedly elevated in the absence of ACS. Electrical disturbances then led to an increased PVC burden and CAVB. He eventually developed sustained VT, likely secondary to the triggered activity of early after-depolarizations. This can explain why overdrive pacing with a transvenous pacemaker was so effective.

Most patients treated with potentially cardiotoxic therapies have a baseline echocardiogram with strain, which can later be used to compare and identify structural or functional decline in patients with suspected or confirmed myocarditis. In a study published by Mahmood et al., 51% had a normal left ventricular ejection fraction (LVEF), and 38% of those who developed major adverse cardiovascular events (MACEs) had a normal LVEF [5]. Our patient, with preserved EF, developed an acute decline in cardiac function observed as global hypokinesis, which later began to improve with corticosteroid therapy, supporting the probable diagnosis of ICI-induced myocarditis. The gold standard diagnostic test for myocarditis is an endomyocardial biopsy, which shows lymphocytic infiltration of the myocardium and myocardial conduction system [8]. Biopsy was considered in the case of our patient, but, like in many other cases, patient instability and concurrent use of anticoagulation delayed and prevented the use of this confirmatory test. Another limitation of biopsies is the possibility of obtaining a false-negative result due to patchy infiltration and sampling errors. In these cases, diagnosis can also be supported by cardiac imaging, such as an MRI, which is the most important non-invasive cardiac imaging modality for the evaluation of myocarditis. Typical imaging findings include myocardial edema and late gadolinium enhancement. However, it is not widely available. Our patient’s clinical instability made transfer for a cardiac MRI unfeasible.

## Conclusions

ICIs are on the rise as therapies for various cancer treatments, and their side effects can often be fatal. Myocarditis induced by ICIs is a rare but potentially lethal complication, and diagnosis is challenging. This can have a detrimental impact on patients' outcomes when appropriate treatment is delayed. It is of utmost importance to recognize the early cardiotoxic manifestations of these medications and commence rapid treatment, which can be life-saving.
